# Synthetic circular miR-21 RNA decoys enhance tumor suppressor expression and impair tumor growth in mice

**DOI:** 10.1093/narcan/zcaa014

**Published:** 2020-07-31

**Authors:** Simon Müller, Alice Wedler, Janina Breuer, Markus Glaß, Nadine Bley, Marcell Lederer, Jacob Haase, Claudia Misiak, Tommy Fuchs, Alina Ottmann, Tessa Schmachtel, Lyudmila Shalamova, Alexander Ewe, Achim Aigner, Oliver Rossbach, Stefan Hüttelmaier

**Affiliations:** Institute of Molecular Medicine, Section for Molecular Cell Biology, Faculty of Medicine, Martin Luther University Halle-Wittenberg, 06120 Halle, Germany; Institute of Molecular Medicine, Section for Molecular Cell Biology, Faculty of Medicine, Martin Luther University Halle-Wittenberg, 06120 Halle, Germany; Institute of Biochemistry, Faculty of Biology and Chemistry, Justus Liebig University of Giessen, 35392 Giessen, Germany; Institute of Molecular Medicine, Section for Molecular Cell Biology, Faculty of Medicine, Martin Luther University Halle-Wittenberg, 06120 Halle, Germany; Institute of Molecular Medicine, Section for Molecular Cell Biology, Faculty of Medicine, Martin Luther University Halle-Wittenberg, 06120 Halle, Germany; Institute of Molecular Medicine, Section for Molecular Cell Biology, Faculty of Medicine, Martin Luther University Halle-Wittenberg, 06120 Halle, Germany; Institute of Molecular Medicine, Section for Molecular Cell Biology, Faculty of Medicine, Martin Luther University Halle-Wittenberg, 06120 Halle, Germany; Institute of Molecular Medicine, Section for Molecular Cell Biology, Faculty of Medicine, Martin Luther University Halle-Wittenberg, 06120 Halle, Germany; Institute of Molecular Medicine, Section for Molecular Cell Biology, Faculty of Medicine, Martin Luther University Halle-Wittenberg, 06120 Halle, Germany; Institute of Biochemistry, Faculty of Biology and Chemistry, Justus Liebig University of Giessen, 35392 Giessen, Germany; Institute of Biochemistry, Faculty of Biology and Chemistry, Justus Liebig University of Giessen, 35392 Giessen, Germany; Institute of Biochemistry, Faculty of Biology and Chemistry, Justus Liebig University of Giessen, 35392 Giessen, Germany; Department of Clinical Pharmacology, Rudolf Boehm Institute for Pharmacology and Toxicology, Faculty of Medicine, Leipzig University, 04107 Leipzig, Germany; Department of Clinical Pharmacology, Rudolf Boehm Institute for Pharmacology and Toxicology, Faculty of Medicine, Leipzig University, 04107 Leipzig, Germany; Institute of Biochemistry, Faculty of Biology and Chemistry, Justus Liebig University of Giessen, 35392 Giessen, Germany; Institute of Molecular Medicine, Section for Molecular Cell Biology, Faculty of Medicine, Martin Luther University Halle-Wittenberg, 06120 Halle, Germany

## Abstract

Naturally occurring circular RNAs efficiently impair miRNA functions. Synthetic circular RNAs may thus serve as potent agents for miRNA inhibition. Their therapeutic effect critically relies on (i) the identification of optimal miRNA targets, (ii) the optimization of decoy structures and (iii) the development of efficient formulations for their use as drugs. In this study, we extensively explored the functional relevance of miR-21-5p in cancer cells. Analyses of cancer transcriptomes reveal that miR-21-5p is the by far most abundant miRNA in human cancers. Deletion of the *MIR21* locus in cancer-derived cells identifies several direct and indirect miR-21-5p targets, including major tumor suppressors with prognostic value across cancers. To impair miR-21-5p activities, we evaluate synthetic, circular RNA decoys containing four repetitive binding elements. In cancer cells, these decoys efficiently elevate tumor suppressor expression and impair tumor cell vitality. For their *in vivo* delivery, we for the first time evaluate the formulation of decoys in polyethylenimine (PEI)-based nanoparticles. We demonstrate that PEI/decoy nanoparticles lead to a significant inhibition of tumor growth in a lung adenocarcinoma xenograft mouse model via the upregulation of tumor suppressor expression. These findings introduce nanoparticle-delivered circular miRNA decoys as a powerful potential therapeutic strategy in cancer treatment.

## INTRODUCTION

MicroRNAs (miRNAs, miRs) are broadly conserved small (20–25 nt), non-coding RNAs (ncRNAs), which inhibit gene expression by inducing the degradation and/or suppressing the translation of target mRNAs (1,2). Consistent with a plethora of mRNA targets, miRNAs have been implicated in the control of various biological and pathological processes, including the development and progression of cancer (3). Oncogenic miRNAs (oncomiRs) are generally upregulated in cancer and inhibit the expression of tumor suppressor-encoding mRNAs. The opposite is considered for tumor-suppressive miRNAs. The duplicity of targeting both types of mRNAs, oncogenic or tumor suppressive, by a single miRNA is one reason why therapeutic approaches are proceeded with caution (4). However, members of the let-7 miRNA family are prominent and well-described examples of largely tumor-suppressive miRNAs, inhibiting mRNAs encoding major oncogenes like RAS family members or HMGA2 (5). In contrast, miR-21 is one of the earliest identified oncomiRs associated with proliferation and invasion during all stages of carcinogenesis. In a large-scale miRNome analysis of 540 tumor samples, miR-21 has been shown to be the most commonly upregulated miRNA in solid human carcinoma, including lung cancer (6). This was confirmed for ovarian (7), thyroid (8), liver (9) and various other types of cancer. The oncogenic role of miR-21 was largely attributed to the inhibition of tumor suppressors, most notably PTEN (10) and PDCD4 (11). MiR-21-directed inhibition of these was proposed to promote the proliferation and metastatic potential of various cancer-derived cells. Its broad upregulation and highly conserved oncogenic properties, e.g. demonstrated for the inhibition of PDCD4 in a variety of cancers (12–17), suggest miR-21 as a prime target for RNA-based therapeutic approaches. However, despite a variety of clinical trials evaluating therapeutic inhibition of miRNA by antagomiRs, the silencing of miR-21 is only considered for the treatment of Alport syndrome (18). In recent studies, artificial circular RNAs were developed to sequester mature miRs resulting in impaired biological function (19,20). Sequestration of HCV-promoting miR-122 by an artificial circular RNA decoy inhibited viral protein synthesis in HCV cell culture systems (19). The transfection of a circular RNA sequestering miR-21 in gastric cancer cells led to increased apoptosis and globally reduced protein synthesis (20). However, in-depth analyses of affected tumor suppressor genes suitable for monitoring therapeutic efficacy, dose dependency and *in vivo* suitability of synthetic circular miR-21-directed decoys are still lacking (21).

## MATERIALS AND METHODS

### Plasmids and cloning

Plasmids including cloning strategies, oligonucleotides for annealing and restriction sites are summarized in Supplementary Table S9. All plasmids were verified by sequencing.

### Cell culture and transfections

A549 (ATCC, RRID: CVCL_0023), H1975 (ATCC, RRID: CVCL_1511), ES-2 (ATCC, RRID: CVCL_3509), Huh-7 (ATCC, RRID: CVCL_0336), C643 (CLS, RRID: CVCL_5969) and HEK293T/17 (ATCC, RRID: CVCL_1926) were cultured in Dulbecco’s modified Eagle medium supplemented with 10% fetal bovine serum at 37°C and 5% CO_2_. The transfection of cells with DNA or decoys was performed using Lipofectamine 3000 (Thermo Fisher Scientific) according to manufacturer’s protocols. Lentiviral particles were generated as previously described (22). Lentiviral transductions were achieved at 10 MOI (multiplicity of infection). For luciferase reporter analyses, 1 × 10^5^ cells were transfected with 100 ng pmirGLO plasmids, as described (22). For CRISPR/Cas9-mediated genomic deletions, 5 × 10^5^ cells were transfected with 1 μg Cas9-encoding plasmid and two sgRNA-encoding plasmids (500 ng each). Plasmids are summarized in Supplementary Table S9. For circular RNA stability analysis, 3 × 10^6^ ES-2 cells were transfected with 750 ng of either circular or linear sponge RNAs by electroporation (GenePulser Xcell; Bio-Rad). Harvested cells were reconstituted in Cytomix (120 mM KCl, 0.15 mM CaCl_2_, 10 mM K_2_HPO_4_/KH_2_PO_4_, pH 7.6, 25 mM HEPES, 2 mM EGTA, 5 mM MgCl_2_, supplemented by 2 mM ATP and 5 mM glutathione directly before use) to 6 × 10^6^ cells/ml. Electroporation was performed with 500 μl cell suspension in 4 mm cuvettes (Sigma) with the following settings: square wave, 270 V, 20 ms, single pulse. A total of 5 × 10^5^ transfected cells were seeded per well in six-well plates.

### Generation of *MIR21* knockouts

Genomic deletions in the *MIR21* locus by CRISPR/Cas9 were achieved by transfection of two sgRNA-encoding plasmids (psg_RFP_miR-21_for, psg_RFP_miR-21-rev, expressing RFP) and the Cas9-encoding plasmid (pcDNA_Cas9-T2A-GFP, expressing GFP). Forty-eight hours post-transfection, single RFP- and GFP-positive cells were seeded by using a FACS Melody sorter (BD Biosciences). The deletion of ∼200 nt in the locus was verified by PCR on isolated gDNA of single-cell clones and sequencing of amplicons. Plasmids and PCR primer are summarized in Supplementary Table S9.

### Cell proliferation, spheroid growth and invasion, and anoikis resistance assays

Cell proliferation was determined in 2D and spheroid culture systems. A total of 1 × 10^3^ cells were seeded in standard (2D) or round-bottom ultra-low attachment (Corning, spheroid) 96-well plates. Spheroid formation was induced by centrifugation at 300 × *g* for 3 min. Cell confluency and spheroid growth were monitored for 5 days using an IncuCyte S3 system (Sartorius) with 4× (2D, whole well scan) or 10× magnification (spheroid). Confluence masks were generated by using the IncuCyte analysis software. CellTiter Glo (Promega) was used to determine cell viability according to manufacturer’s protocols. For anoikis resistance assays, 1 × 10^3^ cells were seeded in flat-bottom ultra-low-attachment plates (Corning) and cultured in media supplemented with 1% fetal bovine serum for 5 days. Colony formation was determined by bright-field microscopy (IncuCyte S3, 4× magnification) and cell viability by CellTiter Glo. Invasion assays were performed by monitoring tumor cell infiltration in Matrigel. Pre-formed spheroids of 1 × 10^3^ cells were embedded in invasion matrix (Trevigen; 6 mg/ml) by centrifugation at 300 × g for 3 min. Infiltration was monitored for 24 h using an IncuCyte S3 system. The relative invasive area and the invasive front were determined by using the IncuCyte analysis software.

### Cell cycle analyses

For cell cycle analyses, A549 cells were harvested using trypsin and fixed overnight in 70% ethanol at −20°C. DNA staining was performed using propidium iodide (Miltenyi Biotec; dilution 1:1000) at 37°C for 30 min in phosphate-buffered saline supplemented with RNase A (Sigma-Aldrich; 2 μg/ml). The DNA content was measured by flow cytometry using a MACS Quant Analyzer (Miltenyi Biotec) and analyzed using FlowJo software.

### Animal handling and xenograft analyses

Immunodeficient athymic nude mice (FOXN1^nu/nu^) were hold according to the guidelines of the Martin Luther University and the University of Leipzig. Local ethical committees granted permissions.

For subcutaneous xenograft studies, 2.5 × 10^5^ control or miR-21 knockout A549 cells, expressing iRFP by lentiviral transduction, were harvested in media and 50% Matrigel (ECM gel from Engelbreth–Holm–Swarm murine sarcoma; Sigma-Aldrich). Cell suspensions were injected into the left flank of 6-week-old nude mice, obtained from Charles River. Chlorophyll-free food *ad libitum* was used to avoid noise in iRFP imaging using a Pear Trilogy System (LI-COR). Tumor growth and volumes were weekly measured and monitored by near-infrared imaging upon isoflurane anesthesia. Volumes were calculated using the following formula: 0.52 × *L*_1_ × *L*_2_ × *L*_3_. Experiments were terminated when first tumors reached a maximal diameter of 1.5 cm. Tumors were excised to determine volume and weight.

### Luciferase reporter assays

Complementary miRNA sequences or 48-nt regions of 3′-UTRs were cloned in the pmirGLO plasmid (Promega). Dual-GLO reporter analyses were performed according to manufacturer’s instructions. Firefly and Renilla luciferase activities were determined 48 h post-transfection of reporters by using a GloMax Explorer Microplate Reader (Promega). Firefly activities were normalized to Renilla activities and to reporters with a minimal 3′-UTR (MCS), as previously described (22).

### RNA isolation and RT-qPCR

Total RNA was isolated by using TRIzol (Thermo Fisher Scientific) according to manufacturer’s instructions. Reverse transcription and quantitative PCR analyses on a Light Cycler 480 II (Roche) were performed as previously described (23). Primers were selected using Primer-BLAST (www.ncbi.nlm.nih.gov/tools/primer-blast/). Genes and sequences are summarized in Supplementary Table S9. The ΔΔ*C*_t_ method was used to determine relative RNA levels.

### Northern blot

Infrared northern blotting of miRNAs and ncRNAs was essentially performed as described before (22). In brief, 4 μg of TRIzol-purified total RNA was separated in a 15% denaturing TBE–urea gel and transferred onto nylon membranes (Roche). After cross-linking (150 mJ/cm^2^), DY782- or DY682-labeled probes (MWG Biotech) were hybridized in PerfectHyb Plus (Sigma-Aldrich) at 20°C for 1 h and monitored using an Odyssey Scanner (LI-COR). Sequences of probes are indicated in Supplementary Table S9.

### RNA sequencing and differential gene expression

Small RNA-seq libraries were prepared by using 50 ng of total RNA, isolated from parental cell lines, as input and the NEXTflex Small RNA Library Prep Kit v3 (Bio Scientific) or by Novogene (Hong Kong). Sequencing was performed on an Illumina HighSeq platform at the Deep Sequencing Group (TU Dresden) or Novogene. For mRNA-seq libraries, polyA-RNA was enriched using oligo(dT) beads. Generation of libraries and sequencing were performed by Novogene on an Illumina HiSeq platform. RNA-seq and miRNA-seq data were analyzed as described previously (22,23). RNA-seq data of the TCGA cohorts were obtained from the GDC portal (https://portal.gdc.cancer.gov).

### Gene set enrichment analyses

Gene set enrichment analyses (GSEA) were performed using the GSEA software (v3.0) (24) with MSigDB (v7.0) gene sets for Hallmark pathways. For the generation of pre-ranked lists, protein-coding genes were ranked according to the correlation coefficient with miR-21 in TCGA lung adenocarcinoma (LUAD) RNA-seq data or the fold change determined upon miR-21 knockout by RNA-seq in A549 cells. Note that TCGA-derived data do not provide miR-21-5p/-3p distinguished information. For correlation analyses of protein-coding genes and miR-21, matching miRNA-seq and mRNA-seq information for each patient of the TCGA LUAD cohort was considered. Data sets were log_2_(RP(K)M + 1)-transformed and the Pearson correlation coefficient (*R*) was determined. Genes were ranked from positive to negative *R* values.

### MicroRNA–target predictions

For the prediction of miR-21-5p target genes, miRWalk v2.0 (http://zmf.umm.uni-heidelberg.de/apps/zmf/mirwalk2/) (25) was used. The following databases were considered for targeting 3′-UTRs of transcripts: miRWalk, miRDB, PITA, MicroT4, miRMap, RNA22, miRanda, miRNAMap, RNAhybrid, miRBridge, PICTAR2 and Targetscan. Prediction scores are indicated in Supplementary Table S6.

### Western blot

Infrared western blotting analyses were performed as previously described (22). In brief, cells were harvested by scraping and total protein was extracted by using lysis buffer (50 mM Tris–HCl, pH 7.4, 50 mM NaCl, 2 mM MgCl_2_, 1% SDS) supplemented with protease inhibitor cocktails (Sigma-Aldrich). Proteins were separated in a NuPAGE 4–12% Bis-Tris gel (Thermo Fisher Scientific) and transferred to an Amersham Protran membrane (GE Healthcare). Protein expression was determined by using specific primary and fluorescence-coupled secondary antibodies and an infrared Odyssey Scanner (LI-COR). Antibodies are summarized in Supplementary Table S9.

### Circular RNA production

Artificial circular RNAs were designed and produced as described in (19,26). In brief, a plasmid backbone flanked by an EcoRI restriction site containing a T7 promoter, a stem–loop, a constant region and an XbaI restriction site was used to insert a double-stranded 5′-phopsphorylated DNA oligonucleotide (Sigma-Aldrich) containing four miRNA-21 binding sites or control sequences (see Supplementary Figure S4). For *in vitro* transcription, a DNA template was excised using EcoRI, gel purified and 200 ng of the DNA template was used in a 1× *in vitro* transcription using HiScribe T7 High Yield RNA Synthesis Kit (NEB), according to the manufacturer’s instructions for short transcripts. Notably, every transcription reaction was supplemented with a 10-fold molar excess of guanosine monophosphate (Sigma-Aldrich). This results in ∼90% of transcripts with a 5′-monophosphate serving to facilitate circularization. The transcription reactions were upscaled 5-fold for production of large quantities of circRNA. DNA templates were digested by RQ1 DNase (Promega). Transcripts were purified by phenol extraction and precipitation, followed by removal of free nucleotides by gel filtration (mini Quick Spin RNA Columns; Roche). To enhance annealing of the stem–loop structure to favor intra- over intermolecular ligation, transcripts were heated to 95°C and cooled to room temperature over 20 min in a thermocycler in the presence of 10 mM Tris–HCl, pH 7.5, and 50 mM NaCl in 100–200 μl. The circularization protocol was modified from (27). Circularization reactions were conducted in 250 or 500 μl volume using T4 RNA ligase 1 (Thermo Scientific or NEB), as described (19). One percent of the total ligation reaction was analyzed on 5%, 6%, 7% or 8% analytical polyacrylamide–urea gel by ethidium bromide staining. Remaining 99% of the total ligation reaction was loaded on a 6% or 8% preparative polyacrylamide–urea gel to excise circular and linear monomer RNAs. The latter were eluted in 1× proteinase K buffer containing 1% SDS for 1 h at 50°C. The elution volume varied between 0.8 and 8 ml depending on the circularization efficiency and therefore on the size of the gel area excised. Eluted RNAs were purified by phenol extraction and precipitation and dissolved in an appropriate amount of RNase-free water. Remaining gel fragments were removed by Costar Spin-X centrifuge tube filters (Corning). The circular RNA production and purification procedure is documented in Supplementary Figure S4B and E. To prove circularity, the RNA preparations were treated using the exonuclease RNase R as described before (26).

### RNA affinity purification

*In vitro* and *in vivo* RNA affinity purification was performed using NeutrAvidin Agarose Beads (Thermo Fisher Scientific). Beads were blocked overnight at 4°C with bovine serum albumin (BSA), tRNA and glycogen (blocking buffer: 4 mM HEPES, pH 7.5, 0.2 mM DTT, 2 mM MgCl_2_, 20 mM KCl, 0.002% NP-40, 0.2 mg/ml tRNA, 1 mg/ml BSA, 0.2 mg/ml glycogen).

For *in vitro* affinity purification, 25 μl packed beads were washed [wash buffer (WB): 20 mM HEPES, pH 7.5, 1 mM DTT, 10 mM MgCl_2_, 0.01% NP-40, 150 or 600 mM KCl] and incubated with 800 fmol biotinylated circular RNA in 800 μl WB-600 for 30 min at room temperature. After washing, beads were incubated with ES-2 cell extract lysed using 4-fold excess RIPA buffer (50 mM Tris–Cl, pH 7.4, 1% NP-40, 0.1% SDS, 150 mM NaCl, 5 mM EDTA, 1× HALT protease inhibitor; Thermo Fisher Scientific) for 1 h at 37°C.

For *in cellulo* affinity purification, 1.6 × 10^7^ A549 cells were transfected with 16 pmol biotinylated circular RNA by using Lipofectamine 2000 Reagent (Thermo Fisher Scientific) and harvested 3 h post-transfection. A549 cellular extracts was generated using 4-fold excess RIPA buffer as described above, and incubated with 50 μl blocked NeutrAvidin Agarose Beads (Thermo Fisher Scientific) for 1 h at room temperature.

For both *in vitro* and *in vivo* RNA affinity purification, beads were washed (4× WB-600, 1× WB-150) afterward and RNA was isolated using TRIzol. Isolated RNA was analyzed by small RNA northern blot using 15% polyacrylamide gels, EDC cross-linking (28), Digoxigenin-LNA-miR-21 probes (Qiagen) and visualized by DIG-Fab-Fragment/CDP-Star-based detection (Sigma-Aldrich).

### Circular RNA stability

For cellular decay analyses, the first time point was represented by directly harvested input material. For other time points (8, 24, 48 and 72 h), medium was exchanged 8 h post-electroporation. Total RNA was isolated by TRIzol and 50% of isolated RNA was analyzed by 7% denaturing polyacrylamide gel electrophoresis. Northern blotting was performed by transfer to nylon membrane (GE Healthcare) and hybridization at 59°C with an internally labeled *in vitro* transcribed ^32^P-riboprobe (71 nt) directed against the constant region of the linear and circular RNAs. In addition, as an internal control, hybridization with a ^32^P-labeled riboprobe against the U1 snRNA (163 nt) was performed. Results were visualized by Typhoon FLA 9500-based phosphorimaging (GE Healthcare). Quantification was conducted using ImageQuantTL software and normalization to inputs. Half-lives of indicated RNA species were derived by fitting to an exponential decay function using OriginPro8 software (ExpDec1 function).

### PEI complexation of circRNA decoys and analysis of physicochemical complex properties

Circular RNA sponges were complexed with low molecular weight polyethylenimine (PEI) F25-LMW (29), as described previously for small RNAs (30). Briefly, 10 μg RNA was complexed in 5% glucose/10 mM HEPES, pH 7.4, by mixing both components at a PEI:RNA mass ratio of 7.5 prior to incubation for 45 min. When preparing larger amounts, complexes were then aliquoted and stored frozen (31). Complexation efficacy at different PEI amounts was determined by agarose gel electrophoresis using 0.5 μg RNA. The complexes were mixed with 10× loading dye and separated onto a 2% agarose gel prestained with 1× Sybr™ Gold in TAE buffer running buffer. Complex stabilities were measured by a heparin displacement assay. PEI F25/RNA complexes were mixed with increasing amounts of heparin as shown in the figure. After an incubation of 30 min, the samples were analyzed by agarose gel electrophoresis as above.

Zeta potentials and particle sizes of complexes were measured as described previously (32). Briefly, complexes containing 20 μg RNA were diluted to 1.5 ml pure water prior to phase analysis light scattering (PALS) and photon correlation spectroscopy, using a Brookhaven ZetaPALS system (Brookhaven Instruments, Holtsville, NY, USA). The data were analyzed using the manufacturer’s software and applying a viscosity and refractive index of pure water at 25°C. Zeta potentials were measured in 10 runs, with each run containing 10 cycles, and applying the Smoluchowski model. For size determination, the complexes were analyzed in five runs with a run duration of 1 min. Results are expressed as intensity-weighted mean diameter from different experiments. Additionally, sizes were analyzed by nanoparticle tracking analysis (NTA) using a NanoSight LM10 (Malvern) equipped with a 640 nm sCMOS camera, software NTA 3.0 and a circRNA concentration of 1 μg/ml.

### PEI/circRNA-based xenograft treatment

For PEI/circRNA therapy studies, 1 × 10^6^ A549 cells in 150 μl medium supplemented with 50% Matrigel were subcutaneously injected into both flanks of nude mice. When tumor volumes reached sizes between 40 and 110 mm^3^, mice were randomized into negative control and specific treatment groups. PEI-based nanoparticles containing 10 μg miR-21-5p or control decoys were intraperitoneally injected over 6 weeks (days 1, 4, 7, 25 and 39). Tumor volumes were regularly monitored and experiments were terminated after 43 days of treatment. Tumors were excised and flash-frozen in liquid nitrogen upon determining volume and mass.

### Statistics

All experiments were performed at least in biological triplicates as indicated. For equally distributed data sets, a parametric Student’s *t*-test was used for statistical significance. Otherwise, a non-parametric Mann–Whitney test was performed as indicated. For RNA-seq false discovery rates (FDRs) were calculated upon TMM normalization. Overall survival analyses were performed using KMplotter (https://kmplot.com/analysis/) (33). Patients were split by auto select best cutoff. For protein-coding genes, the LUAD cohort (*n* = 865 patients) based on gene chips was considered. For miRNAs, miR power based on the TCGA LUAD cohort (*n* = 513 patients) was used. Log-rank analyses were used for statistical significance of Kaplan–Meier plots.

## RESULTS

### MicroRNA 21 is the most conserved and abundant miRNA in cancer

**Figure 1.**
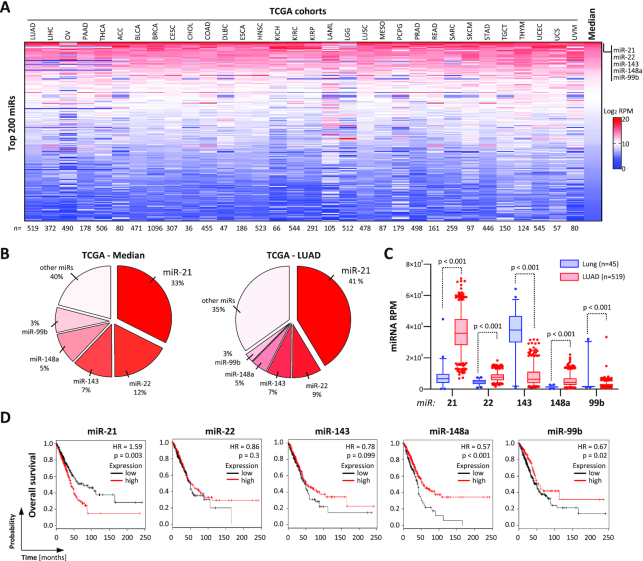
MiR-21-5p is the most abundant oncomiR across cancer. (**A**) Heat map showing the expression (log_2_ RPM) of the 200 most abundant miRNAs in indicated TCGA miRNA-seq data sets. miRNAs are ranked according to the median log_2_ RPM across all data sets. Numbers of patients in each cohort are indicated in the bottom panel. Sequencing data are summarized in Supplementary Table S1. (**B**) Pie charts indicating the percentage distribution of miRNAs in all TCGA (left) and LUAD (right) miRNA transcriptomes. (**C**) Box plots of miRNA expression in lung (blue, *n* = 45) or LUAD (red, *n* = 519) data sets. Statistical significance indicated by *P*-values was determined by the Mann–Whitney test. (**D**) Kaplan–Meier plots of overall survival analyses based on expression (best cutoff) of indicated miRNAs in LUAD patient samples. HR, hazard ratio; *p*, log-rank *P*-value.

The microRNA 21-5p (miR-21) has been reported as a potent oncomiR in various cancers (34,35). Inspection of miRNA expression in 33 TCGA-provided tumor cohorts, including miRNome data of 9891 tumor patients, revealed that miR-21 is the most abundant miRNA with a median percentage of 33% across cancers (Figure 1A and B; Supplementary Table S1). Other miRs with still high, but substantially lower median abundance than miR-21 are miR-22 (12%), miR-143 (7%), miR-148a (5%) and miR-99b (3%). In LUAD, median miR-21 abundance was even further pronounced to 41% and substantially upregulated compared to normal lung tissue (Figure 1B, right panel, and C). Although miRs 22, 148a and 99b were significantly increased as well, their degree of upregulation and total abundance falls way behind miR-21. In agreement with a tumor-suppressive role of miR-143 (36), this miRNA was significantly downregulated in LUAD. Importantly, significant association with adverse prognosis in LUAD was only observed for miR-21 (Figure 1D). For the other most abundant miRs (miRs 22, 148a and 99b), elevated expression, surprisingly, was associated with a rather good prognosis, as expected for miR-143. Collectively, these findings indicated that miR-21 is the most commonly upregulated, abundant miRNA in cancer with reported oncogenic potential. This suggested miR-21 as the prime therapeutic target candidate in cancers and LUAD in particular.

### Deletion of the *MIR21* gene locus impairs LUAD tumor growth

**Figure 2.**
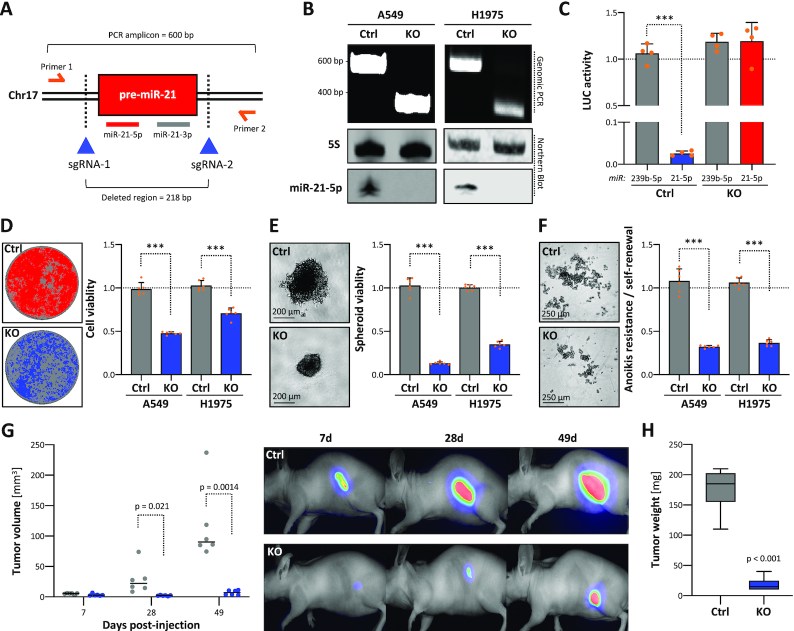
Genomic deletion of *MIR21* in cancer impairs tumor growth. (**A**) Schematic showing the experimental strategy to delete the *MIR21* locus by using Cas9 nuclease and indicated CRISPR guide RNAs (sgRNAs). Primers for PCR on genomic DNA (gDNA) and amplicon/deleted region sizes are indicated. (**B**) Representative PCR analysis on gDNA of parental (Ctrl) and *MIR21*-deleted (KO) A549 and H1975 cells (top panel). Representative northern blot analysis of miR-21-5p expression in Ctrl or KO A549 and H1975 cells (bottom panel). 5S served as a normalization control. (**C**) miRNA reporter analyses in A549 Ctrl or miR-21 KO cells. The activity of indicated miRNAs (cel-miR-239b-5p and hsa-miR-21-5p) was analyzed by using antisense luciferase reporters. Luciferase activities, normalized to a control reporter comprising a minimal 3′-UTR, were determined in four experiments. Viability in 2D (**D**) or 3D spheroid growth (**E**) and anoikis resistance (**F**) analyses of parental (Ctrl) or miR-21 KO A549 and H1975 cells. Representative images of A549 cells are shown (left panels). Viability and anoikis resistance were determined by CellTiter Glo in six median-normalized experiments (right panels). (**G**) Ctrl and miR-21 KO A549 cells expressing iRFP were injected (s.c.) into nude mice (*n* = 6 per condition). Xenograft tumor volumes were measured (left panel) and monitored by near-infrared imaging (right panel) at indicated time points upon injection. (**H**) Box plots showing final tumor mass 49 days post-injection. Statistical significance, indicated by *P*-values, was determined by the Mann–Whitney test: ***P < 0.001.

Previously, the deletion of the *MIR21* gene locus by TALEN and CRISPR/Cas9 technologies revealed a substantially impaired oncogenic potential of HeLa cells and independent, oncogenic roles of miR-21-5p/3p in squamous cell carcinoma stem cells (37,38). In this study, miRNA sequencing in LUAD-derived A549 cells identified an exceedingly high abundance of miR-21-5p, representing ∼68% of all miRNAs (Supplementary Figure S1A; Supplementary Table S7). In contrast, miR-21-3p was substantially less abundant representing only ∼0.08% of the cellular miRNome. To evaluate the oncogenic potency of miR-21-5p in LUAD-derived cells, the *MIR21* locus was deleted in A549 as well as H1975 cells by CRISPR/Cas9 using two sgRNAs (Figure 2A). PCR on genomic DNA and northern blotting confirmed homozygous deletion of *MIR21* and loss of miR-21-5p expression (Figure 2B). In agreement, the repression (∼98%) of luciferase reporter comprising one miR-21-5p complementary targeting site in the 3′-UTR was completely abrogated in *MIR21*-KO cells (Figure 2C; Supplementary Figure S1B). The activity of corresponding miR-21-3p reporters remained largely unaffected in parental cells, supporting the low abundance of this miRNA (Supplementary Figure S1C). This obviously suggested that miR-21-3p activity is largely irrelevant in LUAD-derived cells and that *MIR21*-deleted cells are a suitable model to study the role of miR-21-5p. *MIR21* silencing significantly decreased 2D proliferation, 3D spheroid growth, anoikis resistance and self-renewal in both LUAD-derived cell lines (Figure 2D–F; Supplementary Figure S1D). These findings implied that *MIR21* deletion also impairs tumor growth *in vivo*. This was analyzed in nude mice subcutaneously injected with A549 cells stably expressing iRFP (infrared fluorescent protein) to trace tumor formation. The deletion of *MIR21* significantly impaired tumor growth and resulted in substantially diminished final tumor volume and mass (Figure 2G and H). In summary, these studies indicated that miR-21-5p is a potent oncogenic driver in LUAD tumor models.

### MiR-21-5p is a major inhibitor of tumor suppressor expression promoting tumor cell vitality

**Figure 3.**
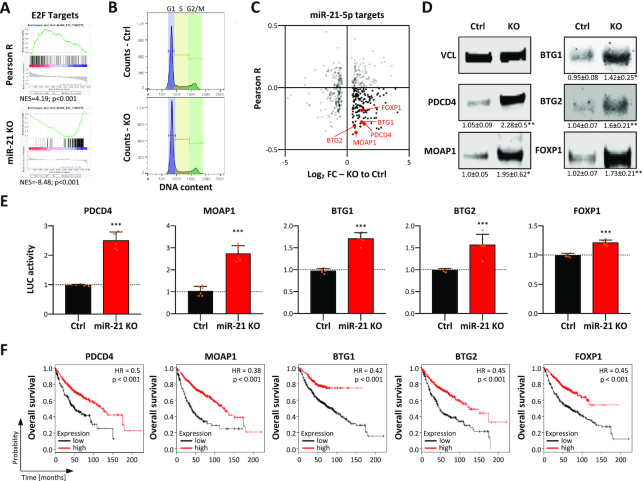
Loss of miR-21 enforces tumor suppressor expression. (**A**) E2F_target GSEA of protein-coding genes ranked by the determined Pearson correlation coefficient determined for their associated expression with miR-21 in the LUAD tumor cohort (upper panel). E2F_target GSEA of genes ranked according to their fold change (FC) of expression in *MIR21*-KO in A549 cells compared to parental cells, as determined by RNA-seq (lower panel). (**B**) Cell cycle phase distribution of A549 Ctrl and *MIR21*-KO cells determined by PI labeling and flow cytometry. (**C**) Scatter plots showing the Pearson correlation of miR-21 expression in LUAD (Pearson *R*) and differential expression of predicted miR-21 target genes (*n* = 444) differentially expressed (FDR < 0.01) upon *MIR21*-KO, as determined in (A). Correlation coefficients were analyzed over 526 LUAD patient samples with matched polyA- and miRNA-seq data sets. MiR-21 target genes were considered when predicted by 5 out of 11 databases analyzed via miRWalk (v2.0). (**D**) Representative western blot analysis of indicated proteins in parental A549 (Ctrl) and *MIR21*-KO cells. VCL served as a loading and normalization control. Relative expression values and standard deviation (SD) were determined in three analyses. (**E**) Luciferase reporter analyses in parental and miR-21 KO A549 cells. The activity of reporters was analyzed by using luciferase reporters comprising 48-nt-long regions of indicated 3′-UTRs including miR-21-5p seed regions. Reporter activities in KO cells were median-normalized to parental cells in six experiments. Statistical significance was determined by Student’s *t*-test: *P < 0.05; **P < 0.01; ***P < 0.001. (**F**) Kaplan–Meier plots of overall survival analyses based on expression (best cutoff) of indicated mRNAs in LUAD patient samples. HR, hazard ratio; *p*, log-rank *P*-value.

Both mature miR-21 ncRNAs, 21-5p as well as 21-3p, have been reported to be involved in the regulation of key oncogenic and tumor-suppressive pathways as well as cancer stem cell properties, in particular self-renewal capacity (38,39). However, in LUAD-derived cells miR-21-3p was found largely irrelevant. Therefore, major cancer-associated pathways controlled by miR-21-5p in LUAD-derived cells were analyzed by monitoring transcriptome changes in *MIR21*-deleted A549 cells by RNA-seq (Supplementary Table S2). GSEA of genes ranked by their fold change of expression upon *MIR21* deletion indicated a striking downregulation of proliferation-associated gene sets, e.g. E2F_target and G2M_checkpoint, as well as other cancer-associated hallmark gene sets like MTORC1_signaling (Figure 3A; Supplementary Table S3). GSEA of genes ranked by their association (Pearson correlation) with miR-21 expression in LUAD (Supplementary Table S4) suggested that gene sets found overall decreased upon *MIR-21* deletion tended to show expression patterns positively associated with miR-21 abundance (Supplementary Table S5). For instance, E2F_target genes were largely downregulated by *MIR21* deletion and were mainly positively associated with miR-21 expression in LUAD, as indicated by an NES (normalized enrichment score) of 4.2 (Supplementary Table S5). Consistent with impaired E2F activity, *MIR21* deletion led to an accumulation of cells in G1 suggesting impaired G1/S transition (Figure 3B; Supplementary Figure S1E). The mRNA levels of positive, e.g. E2F1, CDK2 and CCNE1, but also negative regulators, e.g. E2F5, RB1 and RBL1, of G1/S transition were found largely downregulated upon *MIR21* loss (Supplementary Table S2). This downregulation was associated with marked upregulation of TGFB1 and other facilitators of TGFB signaling like TGFBR2. In view of the substantial downregulation of CDC25A expression and upregulation of SKP2, this suggested that the loss of miR-21 promotes a TGFB signaling-dependent impairment of G1/S transition, as previously proposed (40). To identify key tumor-suppressive factors directly inhibited by miR-21-5p in primary tumors and LUAD-derived cells, the correlation of mRNA expression with miR-21 in LUAD was determined for mRNAs with predicted miR-21-5p target sites that were differentially expressed in A549 cells upon deletion of *MIR21* (Figure 3C; Supplementary Table S6). This identified various validated tumor suppressors upregulated upon *MIR21* deletion. These showed substantial negative association with miR-21 expression in LUAD (Figure 3C, black and red dots). The respective tumor suppressors included the previously reported miR-21-5p targets BTG2 and PDCD4 (14,41,42), as well as BTG1, FOXP1 and MOAP1 (43–45), not previously described as miR-21-5p targets. In agreement with their significantly elevated mRNA abundance in A549 and H1975 *MIR21* knockout cells (Supplementary Figure S2A and B), the protein levels of all five tumor suppressors were upregulated by the loss of miR-21 expression (Figure 3D). Direct miR-21-5p effects were evaluated by testing the regulation of luciferase reporters containing predicted targeting sites with flanking sequences in their 3′-UTRs (Supplementary Figure S2C). The activity of all five tested reporters was significantly increased by *MIR21* loss, indicating the repression of all five tumor suppressors by miR-21-5p in LUAD-derived A549 cells (Figure 3E). Notably, the analysis of overall survival probability revealed the prognostic relevance of all five miR-21-5p regulated tumor suppressors in LUAD. For each of them, a negative correlation with miR-21 expression was observed (Supplementary Figure S2D) and their low abundance was associated with significantly reduced overall survival probability (Figure 3F). Taken together, these findings demonstrated that miR-21-5p is a key inhibitor of tumor suppressors impairing major pro-proliferative oncogenic pathways.

### MIR-21-5p indirectly enhances the expression of oncogenic factors repressed by let-7 miRNAs

**Figure 4.**
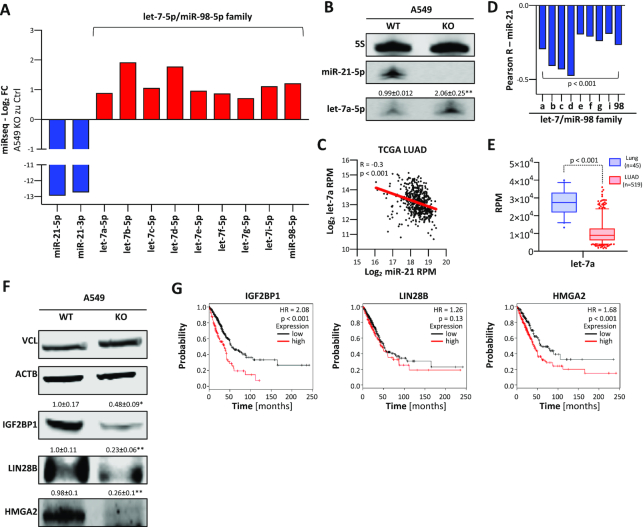
Loss of miR-21 reduces the expression of oncogenic let-7 targets. (**A**) Expression changes of indicated miRNAs in *MIR21*-KO A549 cells, as determined by miRNA-seq. (**B**) Representative northern blot of three analyses for miR-21-5p and let-7a-5p in parental (WT) and *MIR21*-KO A549 cells. 5S served as a loading and normalization control. (**C**) Scatter plots showing the expression of miR-21 and let-7a in TCGA LUAD patients (*n* = 519). Pearson correlation coefficient (*R*) and *P*-value are indicated. (**D**) Pearson correlation coefficients of miR-21 with members of the let-7/miR-98 family in the TCGA LUAD cohort. (**E**) Box plots indicating the expression of let-7a in normal lung tissue (*n* = 45) and LUAD (*n* = 519). Statistical significance was determined by the Mann–Whitney test. (**F**) Representative western blot of three analyses for IGF2BP1, LIN28B and HMGA2 protein expression in parental and *MIR21*-KO A549 cells. VCL and ACTB served as loading and normalization controls. Statistical significance was determined by Student’s *t*-test: *P < 0.05; **P < 0.01. (**G**) Kaplan–Meier plots of overall survival analyses based on expression (best cutoff) of IGF2BP1, LIN28B and HMGA2 mRNAs in LUAD patient samples. HR, hazard ratio; *p*, log-rank *P*-value.

MiRNA-seq confirmed the severe reduction (to ∼38% of parental cells) of total miRNA abundance in A549 cells upon deletion of *MIR21* due to the essential loss of miR-21-5p and -3p (Supplementary Table S7). However, total miRNA abundance remained ∼6% higher than expected (to ∼32% of parental cells) upon *MIR21* deletion, suggesting an upregulation of some miRNAs. Intriguingly, miRNA-seq indicated elevated expression of the complete, tumor-suppressive let-7 miRNA family in *MIR21*-KO cells (Figure 4A and B; Supplementary Table S7). Concomitantly, let-7 miRNA family members like let-7a were significantly downregulated in LUAD and showed an inverse association with miR-21 expression. This suggested that miR-21 interferes with the suppression of oncogenic factors by let-7 miRNAs (Figure 4C–E). In support of this, the loss of *MIR21* was associated with reduced expression of the three major oncofetal let-7-5p target proteins IGF2BP1, LIN28B and HMGA2 (Figure 4F) (5). Notably, these three proteins form an oncogenic triangle antagonizing tumor-suppressive functions of let-7 in cancer cells (46). In LUAD, a pro-oncogenic role of these oncofetal proteins is supported by their significant upregulation and association with reduced survival probability (Figure 4G). LIN28B is a key inhibitor of let-7 expression (47), suggesting that its downregulation upon *MIR21* loss is a key driver of let-7 upregulation. In conclusion, our results indicated that, beyond the direct inhibition of protein-coding tumor suppressors, miR-21 indirectly promotes the expression of pro-oncogenic factors like LIN28B by downregulating miRNAs of the let-7 family expression via mechanisms yet to be characterized in detail.

### MiR-21 is a conserved oncomiR in cancer

**Figure 5.**
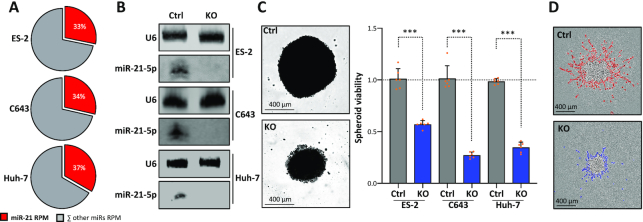
*MIR21*-KO concisely reduces proliferation and invasion in distinct cancer-derived cells. (**A**) Pie charts showing the percentage of miR-21-5p (red) and all other miRNAs in ES-2, C643 and Huh-7 cells, as determined by miRNA-seq. (**B**) Representative northern blot (*n* = 3 experiments) of miR-21-5p in parental and *MIR21*-KO ES-2, C643 and Huh-7 cells. U6 served as a loading control. (**C**) Proliferation of parental and *MIR21*-KO ES-2-, C643- and Huh-7-derived spheroids (*n* = 6 each) was determined by using CellTiter Glo (right). Statistical significance was determined by Student’s *t*-test: ***P < 0.001. Representative images of ES-2 spheroids are shown (left). (**D**) Representative bright-field image (*n* = 3 analyses) of ES-2 spheroid invasion in Matrigel. The invasive front is shown in red (Ctrl, parental) or blue (*MIR21*-KO).

The consistently high abundance of miR-21 expression in human cancers, its validated role in directly inhibiting the expression of conserved tumor suppressors and the indirect enhancement of conserved pro-oncogenic factors suggested that its oncogenic roles are highly conserved as well. This was analyzed by deleting *MIR21* in three additional cancer cell lines: ES-2 (ovarian cancer), C643 (anaplastic thyroid carcinoma) and Huh-7 (hepatocellular carcinoma) cells. In all these cell lines, miR-21-5p was the most abundant single miRNA, as revealed by miRNA-seq (Figure 5A; Supplementary Table S8). Moreover, miR-21-5p showed strong biological activity in all these cancer cell lines, as indicated by the nearly complete repression of luciferase reporters comprising one complementary miR-21-5p targeting site (Supplementary Figure S3A). Deletion of *MIR21* in these cells abolished miR-21-5p expression (Figure 5B). This was associated with substantially impaired cell proliferation (Supplementary Figure S3B), spheroid growth in all three cell lines and markedly reduced Matrigel invasion of ES-2 cells (Figure 5C and D; Supplementary Figure S3C and D). In sum, these findings confirmed the high conservation of miR-21-5p’s oncogenic roles in cancer cells supporting the initial hypothesis that targeting miR-21, specifically miR-21-5p, is likely beneficial in a broad variety of cancers.

### Inhibition of miR-21-5p by circular RNA decoys impairs the oncogenic potential of tumor cells

**Figure 6.**
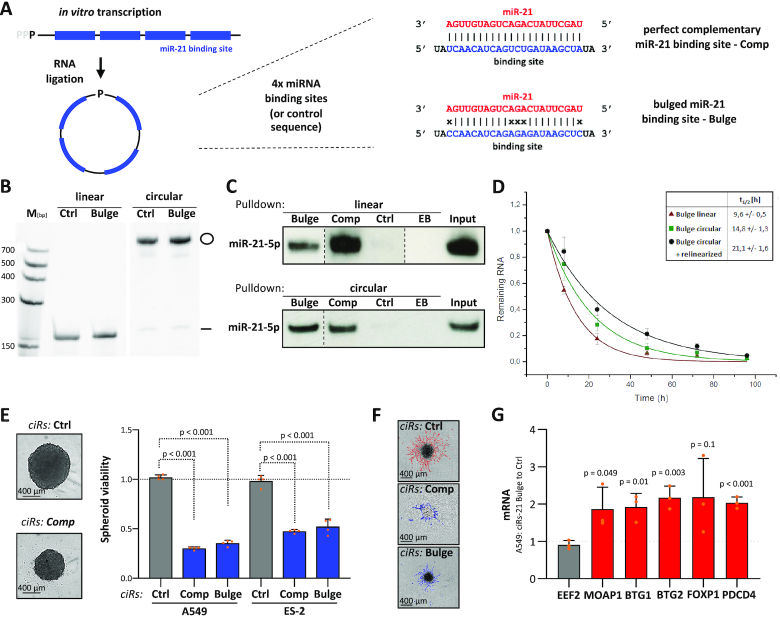
Circular miR-21-5p RNA decoys impair cancer cell proliferation and invasion. (**A**) Schematic showing the synthesis strategy of ciRs containing four miRNA-binding or control sequences. Single, perfectly complementary and bulged miR-21-5p binding sites are depicted (right panel). (**B**) Representative northern blot analysis (*n* = 3 experiments) of linear and circular RNAs before (left) and upon RNA ligation (right). (**C**) Representative northern blot analysis of miR-21-5p associated with linear (top panel) or circular (bottom panel) immobilized miRNA decoys in transfected ES-2 cells. EB, empty beads. (**D**) Decay of linear (red) and circular (green) bulged miR-21-5p decoys was determined by northern blotting upon transfection in ES-2 cells. The biphasic degradation (circular + relinearized) of circular RNA decoys is shown in black. Half-lives of indicated RNA species are indicated (*n* = 3 experiments). (**E**) Proliferation of A549 or ES-2 spheroids was determined by using CellTiter Glo upon transfection with indicated ciRs in four experiments. Representative bright-field images of ES-2 spheroids (left panel). (**F**) Representative bright-field images of ES-2 spheroids invading Matrigel upon transfection of indicated ciRs (*n* = 5 experiments). The invasive fronts are shown in red (Ctrl) or blue (miR-21-5p decoys complementary and bulged). (**G**) RT-qPCR analysis of mRNA levels in A549 cells transfected with bulged miR-21-5p ciRs normalized to Ctrl ciRs. RPLP0 served as a normalization and EEF2 as a negative control in three experiments. Error bars indicate SD. Statistical significance, indicated by *P*-values, was determined by Student’s *t*-test.

Inspired by the efficient miR sponging by naturally occurring circular RNAs (48,49), miR-21-5p-directed circular RNA decoys (ciRs), recently described to disturb gastric carcinoma cell vitality (20), were explored in LUAD-derived cells and 3D cell models. Two miR-21-5p-directed ciRs were generated. Beyond previous studies, exclusively describing decoys containing five bulged miR-21-5p targeting sites, we extended our studies toward shorter decoys with either four perfectly complementary or four bulged miR-21-5p sites (Figure 6A; Supplementary Figure S4A). Upon synthesis of linear RNAs (Supplementary Figure S4B) by *in vitro* transcription as well as circular RNA decoys by subsequent ligation and purification (Supplementary Figure S4C and D), the efficiency of decoys in binding miR-21-5p was evaluated by affinity purification of biotinylated RNA in ES-2 cell lysates (Figure 6B and C). Irrespective of circularization or bulging, all decoys efficiently associated with miR-21-5p. To evaluate the stability of circular versus linear miR-21-5p decoys, their decay in transfected ES-2 cells was monitored by northern blotting (Figure 6D). These studies revealed that circularization substantially increased the intracellular stability of miR-21-5p decoys, as indicated by a >2-fold increase in the half-life of circular decoys (*t*_1/2_ ∼ 21 h) when compared to linear counterparts (*t*_1/2_ ∼10 h). This elevated stability resulted from a two-step decay of circular decoys with a relinearized intermediate (50). In sum, these studies revealed that circularization substantially increased the cellular stability of miR-21-5p decoys and thus suggested ciRs as effective inhibitors of miR-21-5p *in cellulo*. This was evaluated further by monitoring the viability of A549 cells transfected with increasing amounts of negative control versus perfectly complementary circular miR-21-5p decoys. The latter strongly impaired cell viability at low doses (Supplementary Figure S5A). Notably, control decoys did not affect viability at any tested concentration, excluding non-specific RNA effects. The circularity of purified and transfected RNAs was monitored by exonuclease treatment and northern detection (Supplementary Figures S4E and S5B), and interaction of miR-21-5p in the transfected cells was validated by affinity purification of transfected biotinylated circRNAs (Supplementary Figure S5C). To evaluate whether growth inhibition is preserved in 3D cell models and among distinct tumor cells, both types of decoys, perfectly complementary or bulged, were analyzed in LUAD-derived A549 cells and ovarian cancer-derived ES-2 cells. Both types of decoys impaired 3D spheroid growth and 2D proliferation at similar efficiency (Figure 6E; Supplementary Figure S5D). Moreover, they substantially interfered with the Matrigel invasion of ES-2 cells, which could not be tested in A549 cells due to insufficient invasive potential (Figure 6F; Supplementary Figure S5E). Strikingly, the growth of A549- or H1975-derived spheroids was unaffected by transfecting corresponding linear RNAs (Supplementary Figure S5G) indicating the importance of circularization and elevated decoy stability. Decreased spheroid growth upon transfection of circular RNAs was associated with a significant upregulation of all prior evaluated tumor suppressors that had been found repressed by miR-21-5p in A549 and H1975 cells (Figure 6G; Supplementary Figure S5F and H). In conclusion, the presented findings revealed that miR-21-5p decoys show high intracellular stability in tumor-derived cells and effectively impair oncogenic roles of miR-21-5p in tumor cells, irrespective of being perfectly complementary or bulged.

### PEI nanoparticles loaded with circular mir-21-5p decoys impair tumor growth *in vivo*

**Figure 7.**
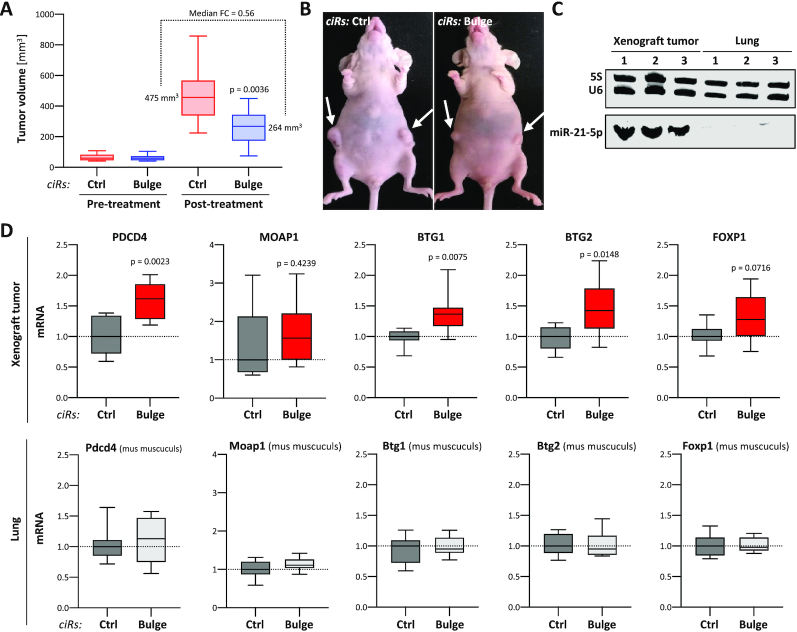
Nanoparticle-delivered miR-21-5p ciRs inhibit tumor growth and enforce tumor suppressor expression in murine LUAD models. (**A**) A549 cells were injected into the left and right flanks of nude mice. Mice were randomized when reaching tumor volume threshold volume (40–110 mm^3^). PEI nanoparticles loaded with Ctrl or bulged miR-21-5p ciRs were i.p. injected five times in 43 days and tumor volume was monitored (post-treatment, Ctrl *n* = 8, bulge *n* = 13). Statistical significance, indicated by *P*-values, was determined by the Mann–Whitney test. (**B**) Representative images of mice treated with Ctrl (left) or bulged miR-21-5p (right) ciRs. Arrows indicate primary tumors. (**C**) Northern blot analysis of miR-21-5p in xenograft tumors and murine lung tissue. 5S and U6 served as loading controls. (**D**) The expression of indicated mRNAs in tumors (*n* = 8, upper panel) and lung tissues (*n* = 7, bottom panel) derived from mice treated with miR-21-5p or control (Ctrl) ciRs is shown by box plots. RNA levels in miR-21-5p ciR-treated samples were normalized to median mRNA abundance in samples treated with Ctrl ciRs, as depicted by dashed lines. Statistical significance, as indicated by *P*-values, was determined using Student’s *t*-test.

The high inhibitory potency and *in cellulo* stability of circular miR-21-5p decoys suggested them as potential candidates for therapeutic intervention, i.e. for inhibiting tumor growth *in vivo*. This was tested in an A549-derived subcutaneous (s.c.) tumor xenograft model in mice. For therapeutic application and *in vivo* delivery, bulged miR-21-5p decoys were formulated in PEI-based nanoparticles. For this, decoys were complexed with the low molecular weight branched PEI F25-LMW (29), as described previously for small RNAs (30). Efficient complexation was achieved already at a low polymer:mass ratio of 2.5, as demonstrated by the absence of the free decoy band in gel electrophoresis (Supplementary Figure S6A). These PEI/decoy complexes were found very stable, requiring 1 unit heparin per 0.2 μg RNA for complex decomposition in heparin displacement assays (Supplementary Figure S6B). Dynamic light scattering revealed complex sizes in the range of ∼130 nm, which was also confirmed by NTA (Supplementary Figure S6C). Finally, PALS revealed a positive zeta potential of ∼20 mV. Taken together, this confirmed the formation of polymeric nanoparticles for decoy delivery and cellular internalization. Previous studies on the *in vivo* biodistribution of PEI/siRNA complexes upon systemic application had identified intraperitoneal (i.p.) injection as more suited over intravenous injection for complex delivery into s.c. tumor xenografts (51). Thus, PEI nanoparticles containing 10 μg miR-21-5p or control decoy were injected five times over 6 weeks (days 1, 4, 7, 25 and 39) in nude mice with A549-derived s.c. tumors. The monitoring of tumor sizes revealed substantially diminished tumor growth in the specific treatment group, becoming obvious already at day 12 of treatment. This trend was further pronounced at the time of termination (day 43), as indicated by a significant and nearly 2-fold decrease in tumor size in miR-21-5p decoy-treated mice (Figure 7A and B). Notably, we observed no obvious side effects upon PEI/decoy nanoparticle treatment in the course of 6 weeks. How decoy treatment influenced gene expression in the lung and s.c. xenograft tumors was analyzed for the five prior identified tumor suppressors targeted by miR-21-5p. Despite isolation of tissue samples 4 days after the last injection of PEI nanoparticles, robust upregulation of all five tumor suppressors was observed in tumors isolated from animals treated with miR-21-5p decoys (Figure 7D, upper panel). In contrast, expression remained essentially unaffected in the lung of animals (Figure 7D, lower panel). Northern blotting clearly indicated that circular and linearized decoys were substantially more abundant in lung tissue (Supplementary Figure S7A). However, consistent with the severe upregulation of miR-21 expression in LUAD compared to healthy lung tissue, northern blotting demonstrated that miR-21-5p levels are substantially lower, actually non-detectable by northern blotting, in mouse lung tissue when compared to xenograft tumors (Figure 7C). These preclinical findings provide strong evidence that the systemic application of circular miR-21-5p decoys delivered by PEI nanoparticles has a high therapeutic potency in impairing tumor growth in mice.

## DISCUSSION

This study demonstrates for the first time the inhibition of the major oncomiR, miR-21-5p, by nanoparticle-delivered circular RNA decoys, leading to efficient impairment of tumor growth in an experimental mouse model. Our results as well as previous findings reported in the literature identify miR-21-5p as a direct or indirect regulator of major tumor suppressor genes. This combined regulation of several targets may provide a substantial advantage for therapies based on miRNA inhibition, with their broader action also addressing the notion of cancer as a pathway disease (52). Concomitantly, miR-21-5p offers a strong predictive value, for example, with regard to overall patient survival, as also seen for the protein tumor suppressors, e.g. BTGs and PDCD4, identified or confirmed here to be regulated by this miRNA. Interestingly, *MIR21* deletion also leads to an upregulation of non-protein tumor suppressors, most prominently an increase of all let-7 miRNA family members. This supports the substantial impact of miR-21 on broad repression of tumor suppressors. Whereas elevated expression of protein tumor suppressors upon *MIR21* deletion is an obvious result of mRNA upregulation, the elevated expression of let-7 miRNAs most likely indicates secondary regulation. This potentially relies on changes in transcription, the processing of precursor RNAs and/or altered turnover of mature or intermediate miRNA precursors. Although requiring further in-depth analyses, upregulation of let-7 miRNAs in *MIR21*-KO cells is expected to largely result from downregulation of the oncogenic RNA-binding protein LIN28B. This destabilizes let-7 miRNAs by oligouridylation (53). Most importantly, however, the substantial abundance across cancers and comprehensive inhibition of tumor suppressors by miR-21-5p indicate this miRNA as a potent and broadly applicable therapeutic strategy in cancer treatment. Such strategies, however, require the development of potent inhibitors as well as their efficient delivery to the desired site of action. Here, we present the first evidence that targeting of miR-21-5p by circular miRNA decoys is effective in distinct tumor cells and impairs tumor growth in xenograft mouse models when delivered by nanoparticles.

Naturally occurring circular RNAs with miR decoy activity have been identified to serve essential roles in controlling gene expression (54–56). In 2018, the first artificial circRNA decoy has been established to target miR-122 that is crucial for hepatic HCV expansion (19). Recently, synthetic circular RNA sponges harboring five bulged miR-21 binding elements have been shown to suppress the proliferation of gastric cancer cells (20), although the circRNA design and production was substantially different from the earlier miR-122/HCV study and this study, as discussed elsewhere (21). In this study, we designed ciRs containing only four either complementary or bulged miR-21-5p binding motifs. The bulged configuration is derived from the natural incorporation of any cellular miRNA into the Ago2 protein. In this complex, positions 10–12 of the miRNA are not required for base pairing with its target sequence (57). These circRNAs proved to be substantially more stable than linear analogues with a cellular half-life of ∼20 h when transfected into cells in this and former studies. This elevated stability apparently results from a biphasic decay mechanism of circular RNA decoys, where the first and rate-limiting step is relinearization by either autohydrolysis or a yet to be determined endonucleolytic activity. In a second step, relinearized ciRs seem to undergo standard RNA decay. Considering the availability of functional miR-21-5p binding sites within the cell after transfection of circular RNA sponges, the detectable amounts of circular and linear RNA sponges have to be added up resulting in an elevated decoy half-life (19). The efficient sequestration of miR-21-5p by these ciRs in cancer cells increased the expression of tumor suppressors targeted by miR-21-5p and, concomitantly, impaired tumor cell vitality. In agreement, the first reported *in vivo* application of ciR-21-loaded PEI nanoparticles efficiently inhibited tumor growth in a subcutaneous LUAD xenograft mouse model. Notably, this was associated with the de-repression of tumor suppressors, otherwise inhibited by miR-21-5p in tumors. While this demonstrates the specificity of this therapeutic intervention on the molecular level, it cannot be excluded that de-repression of other miR-21-5p targets may be involved in the antitumor effects as well, thus adding to the overall efficacy of miR-21-5p inhibition. Notably, although largely accumulated in healthy lungs, the expression of tumor suppressors remained unaffected by ciRs, most likely due to exceedingly low miR-21 levels in healthy lung tissue. This underscores the specificity of the circular miR-21 decoys used here. In a related approach, Wang and colleagues transfected a human adenocarcinoma cell line with circular miR-21 decoys using lipofection before implanting these cells into mice (58). Although this supports the notion of effective inhibition of tumor cell growth by miR-21-5p-directed decoys *in vivo*, the here presented studies present the first evidence that therapeutic delivery of ciRs by nanoparticles impairs growth of preformed tumors.

PEIs have been shown previously to deliver plasmid DNA as well as small RNA molecules *in vitro* and *in vivo* [see e.g. (29,59–62)]. In this study, we extend the use of PEI nanoparticles for the first time toward circRNAs and demonstrate high efficacy. This is particularly noteworthy since several liposomal and polymeric nanoparticle systems, including PEI complexes, have been found rather inefficient for mRNA delivery, which is generally considered more challenging than that of small oligonucleotides [see e.g. (63,64) and references therein]. In the case of PEI, it has been tempting to speculate that poor intracellular complex decomposition may be an underlying reason for this inefficacy, due to long, linear RNA molecules forming more stable complexes based on intramolecular cooperativity in electrostatic PEI/RNA interactions. This study, however, demonstrates the capability of PEIs for efficient *in vivo* delivery and activity of circular RNAs. In combination with its favorable biocompatibility, the low molecular weight PEI F25-LMW thus provides a platform for the therapeutic use of circRNAs as well as for further improvement of nanoparticle properties. This may well include even shorter PEIs and/or their chemical modifications [see e.g. (65)]. It has been controversially discussed whether artificial circular RNAs trigger the innate immune response as RNA viruses (66,67). The Huh-7.5 cell line used in the earlier study is defective in RNA sensory pathways triggered by RIG-I or TLR-3 (19), but this may be relevant in anti-tumor approaches. It remains to be elucidated whether and how circular RNAs are recognized by the cell or in the context of the whole organism. Since RNA recognition largely relies on the detection of RNA ends, circRNAs may avoid innate immunity (67).

In summary, this study provides a proof of principle that circular miRNA decoys are suitable and effective tools for the *in cellulo* as well as *in vivo* inhibition of miR-directed gene silencing. Notably, substantial inhibition was demonstrated for miR-21-5p, the by far most abundant miRNA in cancer cells. This indicates ciRs as easily and cost-effectively produced miR inhibitors, providing high flexibility by rapidly introducing variable miR-binding motifs depending on cellular miRNA expression patterns. Thus, the here presented findings unravel a new, exciting perspective in RNA-based drug development.

## DATA AVAILABILITY

Total RNA-seq as well as small RNA-seq data were deposited at NCBI GEO (GSE148736).

## Supplementary Material

zcaa014_Supplemental_Files
