# Supplementary material for: Synthetic circular miR-21 RNA decoys enhance tumor suppressor expression and impair tumor growth in mice
Source: NAR Cancer. 2020 Jul 31;2(3):zcaa014. doi: 10.1093/narcan/zcaa014 (PMC8210135; doi:10.1093/narcan/zcaa014)

SUPPLEMENTARY FIGURE LEGENDS

Supplementary Figure S1. Loss of miR-21 impairs proliferation of LUAD-derived cancer cells. **(A)** Scatter plot (left panel) showing miRNA expression in two A549 miRNA-seq data sets. Selected miRNAs of Figure 1 are indicated. Pie chart (right panel) indicating the distribution of miR-21-5p/3p and all other annotated miRNAs in A549 cells determined by miRNA-seq. **(B)** miRNA-reporter analyses in H1975 Ctrl or miR-21 KO cells. The activity of the miRNAs (cel-miR-239b-5p and hsa-miR-21-5p) was analyzed as in Figure 1C in four independent experiments. **(C)** Reporter analysis showing the activity of miR-21-3p in A549 and H1975 cells. Analyses were performed as in Figure 2C in four independent experiments. **(D)** Representative images of H1975 Ctrl and *MIR21*-KO cells from Figure 2D-F are shown. **(E)** Fractions of A549 Ctrl and miR-21 KO cells in each cell cycle phase were quantified in five independent analyses. Statistical significance was determined by Student's t-test: *, $p<0.05$; ***, $p<0.001$

Supplementary Figure S2. Tumor suppressor-encoding mRNAs are repressed by miR-21 in LUAD-derived cells. **(A)** Abundance of indicated mRNAs in A549 Ctrl and miR-21 KO cells determined by RNA-seq. Fold changes and p-values derived from 3 independent samples are shown. **(B)** RT-q-PCR analysis of mRNA levels in H1975 miR-21 KO cells normalized to Ctrl cells. RPLP0 served as normalization and EEF2 as negative control. Statistical significance was determined by using Student's t-test. **(C)** Schematic presenting luciferase reporters as in Figure 3E. Luciferase reporters comprise 48nt regions of the indicated 3'UTRs, including miR-21-5p seed regions (highlighted in red). **(D)** Scatter plots showing the expression of miR-21-5p (log_2 RPM) and tumor suppressor-encoding mRNAs (log_2 RPKM) in matched TCGA LUAD patients as in Figure 3C. Pearson correlation coefficient (R) and p-value are indicated. **(E)** Box plots of mRNA expression in lung (red, n=59) or LUAD (blue, n=533) RNA-seq data sets. Statistical significance was determined by using Mann-Whitney test.

Supplementary Figure S3. MiR-21 promotes the proliferation of cancer-derived cells. **(A)** MiRNA-reporter analyses in Ctrl or miR-21 KO ES-2, C643 and Huh-7 cells. Activities of miRNAs (cel-miR-239b-5p and hsa-miR-21-5p) were determined and normalized as in Figure 2C. **(B)** Proliferation in 2D cultures of Ctrl or miR-21 KO ES-2, C643 and Huh-7 cells was determined by CellTiter GLO and median-normalized in six independent experiments. **(C)** Representative images of Ctrl and *MIR21*-KO C643- and Huh-7-derived spheroids as in Figure 5C. **(D)** Quantification of the relative invasion area of Ctrl and *MIR21*-KO ES-2 spheroids from Figure 5D in six independent experiments. Statistical significance was determined by Student's t-test: **, $p<0.01$; ***, $p<0.001$

Supplementary Figure S4. Sequence composition and *in vitro* production of artificial circular RNA decoys targeting miR-21 and control decoys. **(A)** Transcript sequences of circular RNA sponges are indicated. The 5'- und 3'-termini of the transcripts form an 11 nt stem structure (in red), flanked by a 5 nt loop on each end that cannot base-pair. The 5'-stem element is succeeded by a 63 nt constant region that is similar in all circRNAs to serve as a hub for PCR primer and northern probe binding (in blue). The miR-21 sponges contain four miR-21 binding sites (underlined, complementary bases as capital letters), separated by a 4 nt spacer. Sequences are summarized in Supplementary Table S9. **(B)** Linear RNA molecules of three miR-21 decoys differing in the sequence of their miR-21 binding sites (Comp: perfectly complementary miR-21 binding sites; Bulge: bulged miR-21 binding sites; Ctrl: control sequence without miR-21 binding sites) were produced by *in vitro* transcription following the protocol given in (Breuer, Rossbach 2020). The DNA template was removed by DNase treatment. Transcripts were analyzed on a 7% polyacrylamide-urea gel and visualized by ethidium bromide staining. **(C)** *In vitro* transcribed RNAs were ligated using T4 RNA ligase, resulting in a mixture of linear monomers (indicated by a dash), circular monomers (circle), linear dimers (double dash), linear trimers (triple dash) and circular dimers (large circle). To identify circularized miR-21 decoy RNAs the ligation reaction was analyzed using three different polyacrylamide-urea gels with 5, 6 and 7% polyacrylamide, respectively. Note that while the mobility of linear RNAs within polyacrylamide gels is proportionally to the size marker, circular RNAs show a decreased mobility within higher percentage polyacrylamide gels, which is detectable as a size shift comparing 5, 6 and 7% polyacrylamide-urea gels. **(D)** Linear and circular monomers from the ligation reaction in (C) were purified from a preparative 6% polyacrylamide gel, and analyzed as described above. Note that the minor linear monomer band in the circular RNA preparations is derived from stochastically occurring autohydrolysis of purified circular RNA molecules rather than co-purified linear monomer transcripts. **(E)** Purified linear and circular monomer RNA molecules were incubated in the presence or absence of RNase R (indicated by "+" and "-"). Afterwards the reaction was analyzed on a 7% polyacrylamide-urea gel and visualized by ethidium bromide staining. Note that the amount of the minor linear monomeric RNA in the circular RNA reactions is not changed with or without RNase R treatment, indicating that the linear monomer detected here are derived from stochastically occurring autohydrolysis of the circular RNA following RNase R treatment rather than RNase R resistant species.

Supplementary Figure S5. Artificial circular miR-21 RNA sponges reduce cell proliferation and enhance tumor suppressor expression. **(A)** Proliferation in 2D cultures of A549 cells transfected with increasing concentrations of Ctrl or miR-21-5p complementary circular RNA decoys (ciRS). Viability was determined by CellTiter GLO in five independent experiments and normalized to the Ctrl median with 0.002 nM ciRS. **(B)** RNase R treatment of total RNA isolated from A549 cells transfected with 200 ng of

either circular or linear isoforms of the described artificial miR-21 sponges. ~~A549 cells were transfected with 200 ng of circular or linear miR-21 sponges.~~ Total RNA was isolated and incubated in the presence or absence of RNase R (indicated by "+" and "-"). Processed samples were analyzed on a 7% polyacrylamide-urea gel followed by visualization of circular and linear RNAs using Northern blotting. Note that the amount of the minor linear monomeric RNA in the circular RNA reactions remains unaffected by RNase R treatment, indicating that the linear monomer detected here are derived from stochastically occurring autohydrolysis of the circular RNA following RNase R treatment rather than RNase R-resistant species. **(C)** *In vivo* miR-21 affinity purification assay of artificial circular miR-21 sponges. A549 cells were transfected with biotinylated, circular miR-21 decoys. Upon affinity purification of decoys, co-purified RNA was analyzed by 7% and 15% polyacrylamide-urea gels. Decoys (upper panel) and mature miR-21-5p (lower panel) were analyzed by Northern blotting. Note that miR-21-5p is only enriched with circular miR-21 decoys. **(D)** 2D proliferation of A549 and ES-2 cells transfected with 2nM Ctrl or miR-21-5p targeting ciRS. Cell viability was determined by CellTiter Glo in four independent experiments and normalized to the Ctrl median (right panel). Representative bright-field images with overlaying confluence masks of ES-2 cells are shown (left panel). **(E)** Quantification of the relative invasion area of ES-2 spheroids transfected with 2nM Ctrl or miR-21-5p targeting ciRS, as depicted in Figure 6F, was performed in five independent experiments. **(F)** Proliferation of H1975-derived spheroids transfected with 2 nM Ctrl or miR-21 ciRs was determined by CellTiter GLO. Four independent experiments were normalized to the Ctrl median. **(G)** Proliferation of A549- and H1975-derived spheroids transfected with 2 nM Ctrl or miR-21 bulge linear RNAs (liRs) was determined by CellTiter GLO in three independent experiments normalized to the Ctrl median. **(H)** RT-q-PCR analysis of mRNA levels in H1975 cells transfected with bulged miR-21 circular RNA sponges normalized to Ctrl ciRS in three independent experiments. RPLP0 served as normalization and EEF2 as negative control. Statistical significance was determined by Student's t-test: *, $p<0.05$; ***, $p<0.001$

Supplementary Figure S6. Physicochemical characterization of PEI/circRNA complexes, with regard to complexion efficacy **(A)**, complex stability **(B)** and complex properties **(C)**.

Supplementary Figure S7. PEI-loaded Circular RNA sponges are observed in murine lung tissue and A549 xenograft tumors upon intraperitoneal injection. **(A)** After sacrificing the mice, RNA was prepared from A549 Xenograft tumors (top panel) and lung tissues (bottom panel). 10 µg of RNA were separated on an 8% denaturing polyacrylamide urea-gel and detected by Northern blot using *in vitro* transcribed, DIG-labeled riboprobes against the constant region of the ciRS as shown in Figure S4. U1 snRNA served as loading control.

Supplementary Figure S1

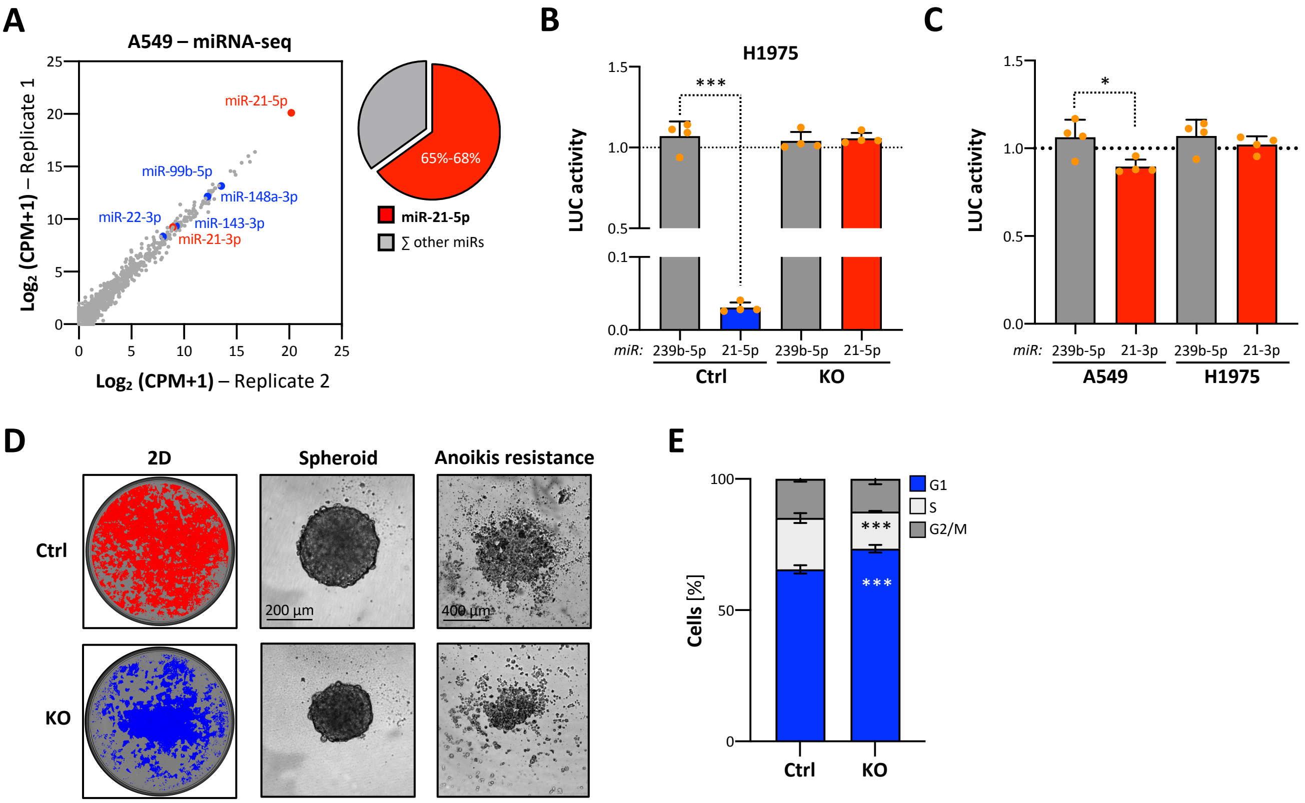

Supplementary Figure S2

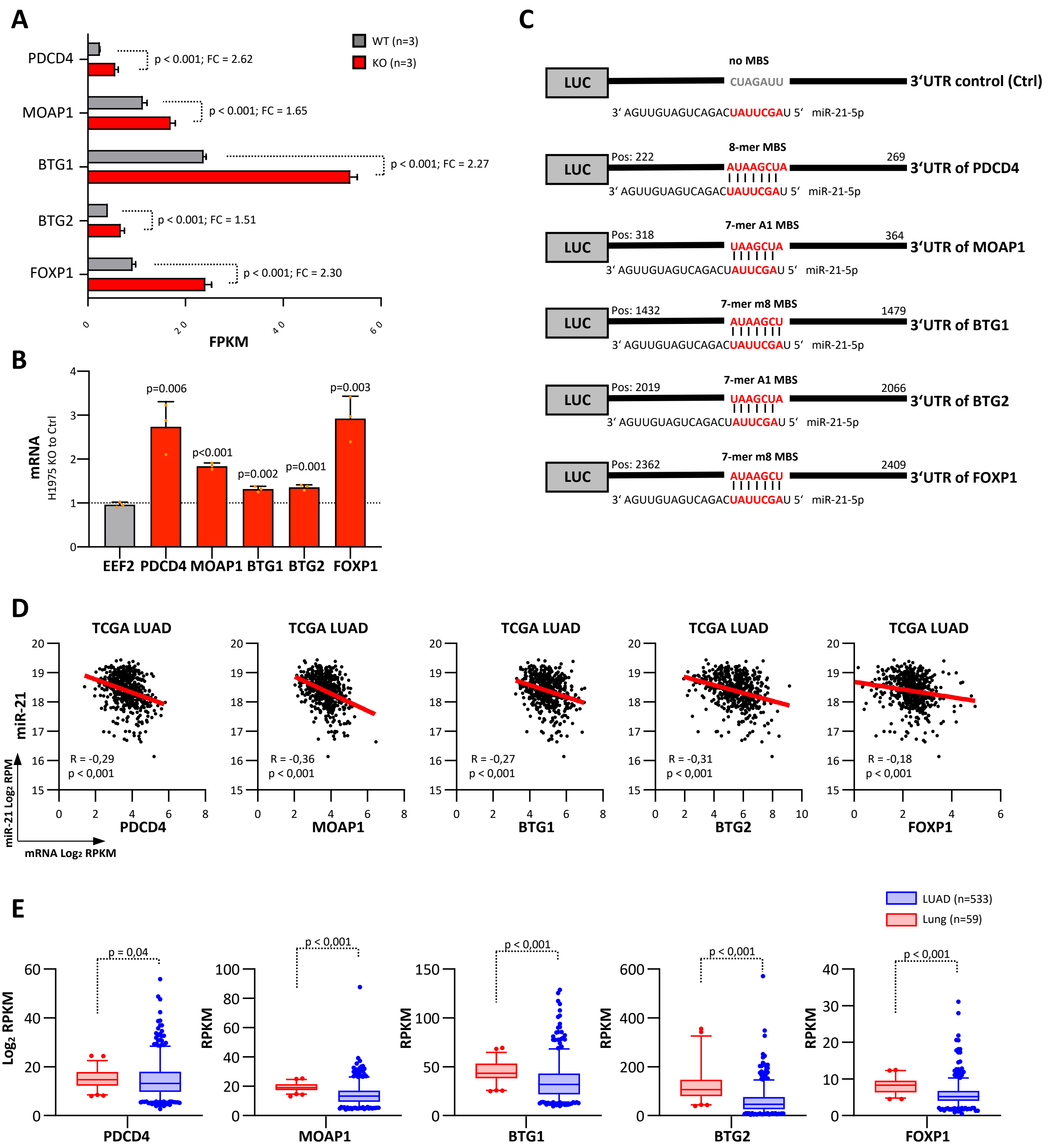

Supplementary Figure S3

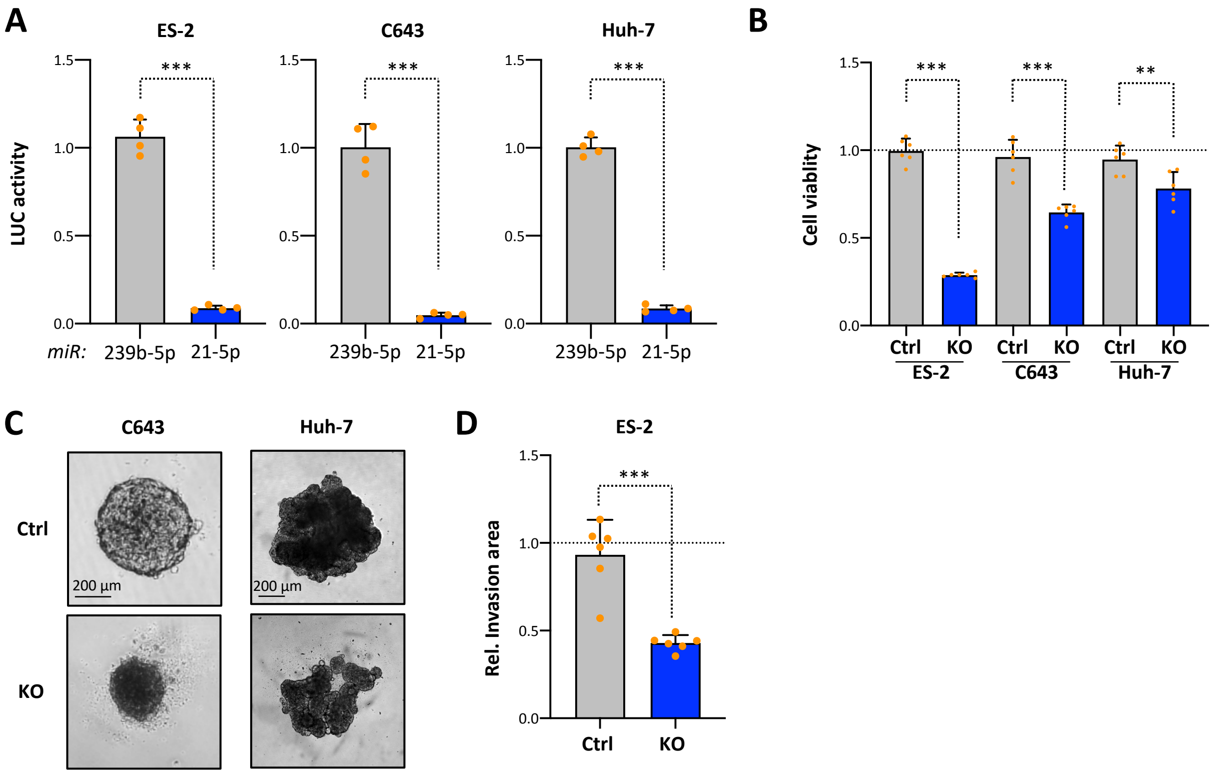

Supplementary Figure S4

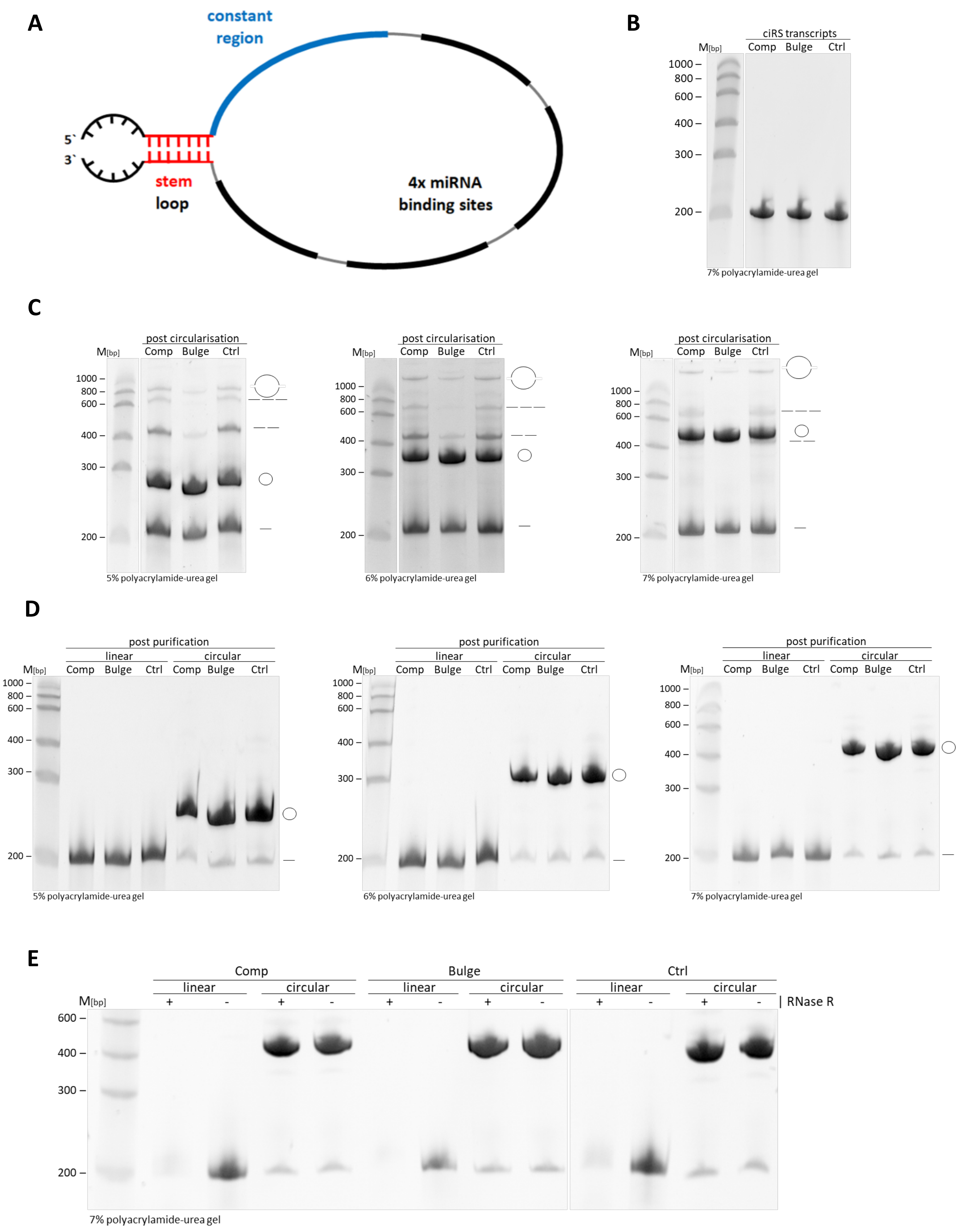

Supplementary Figure S5

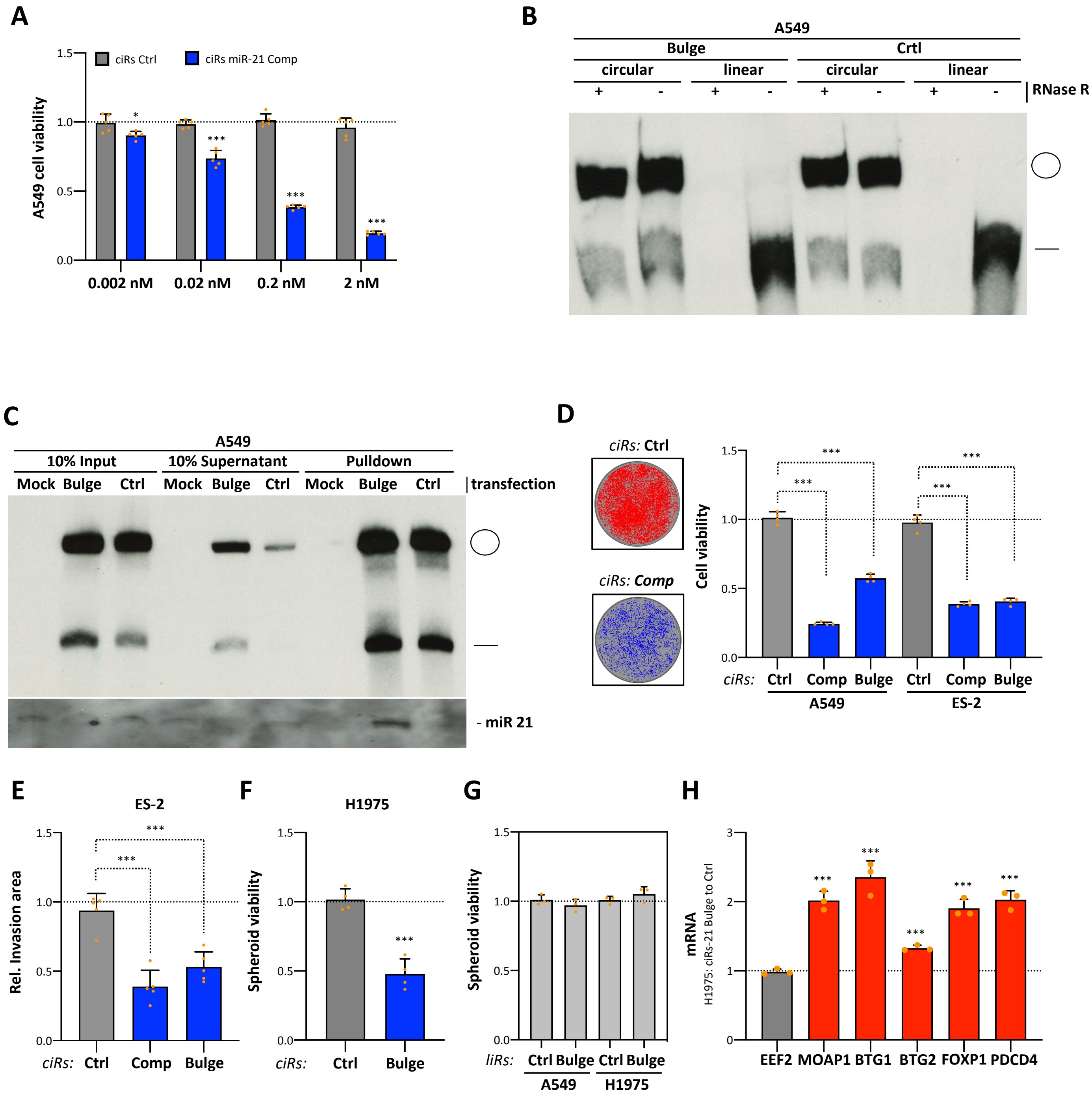

Supplementary Figure S6

A

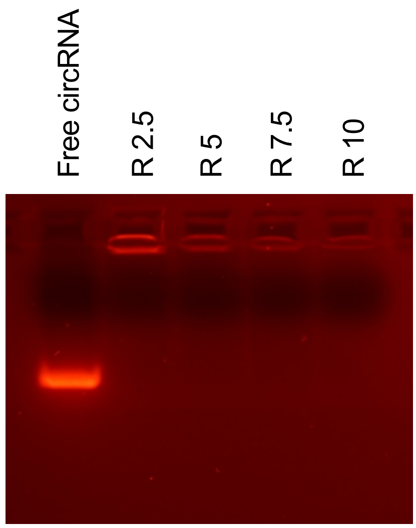

5% Glucose, 10mM HEPES

B

Units heparin/0.2µg circRNA

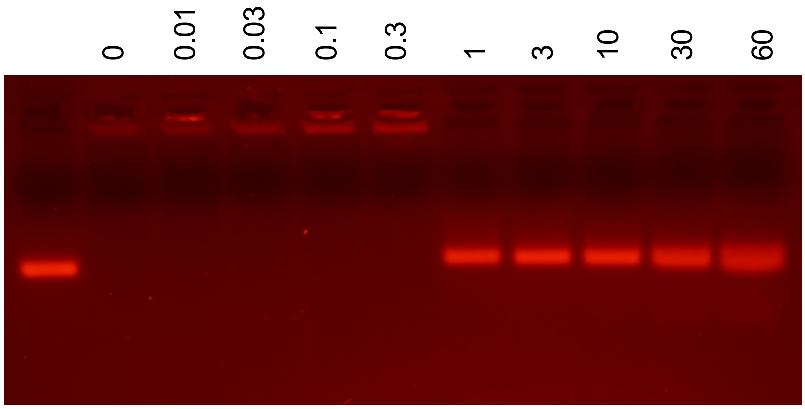

C

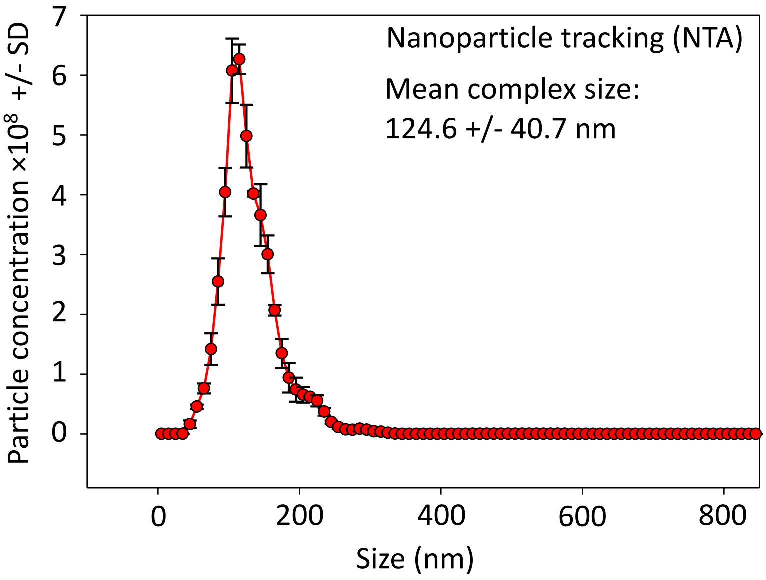

	DLS	NTA
Mean diameter	133.8 +/- 1.8 nm	124.6 +/- 40.7 nm

Zeta potential	19.99 +/- 1.7 mV

Supplementary Figure S7

A

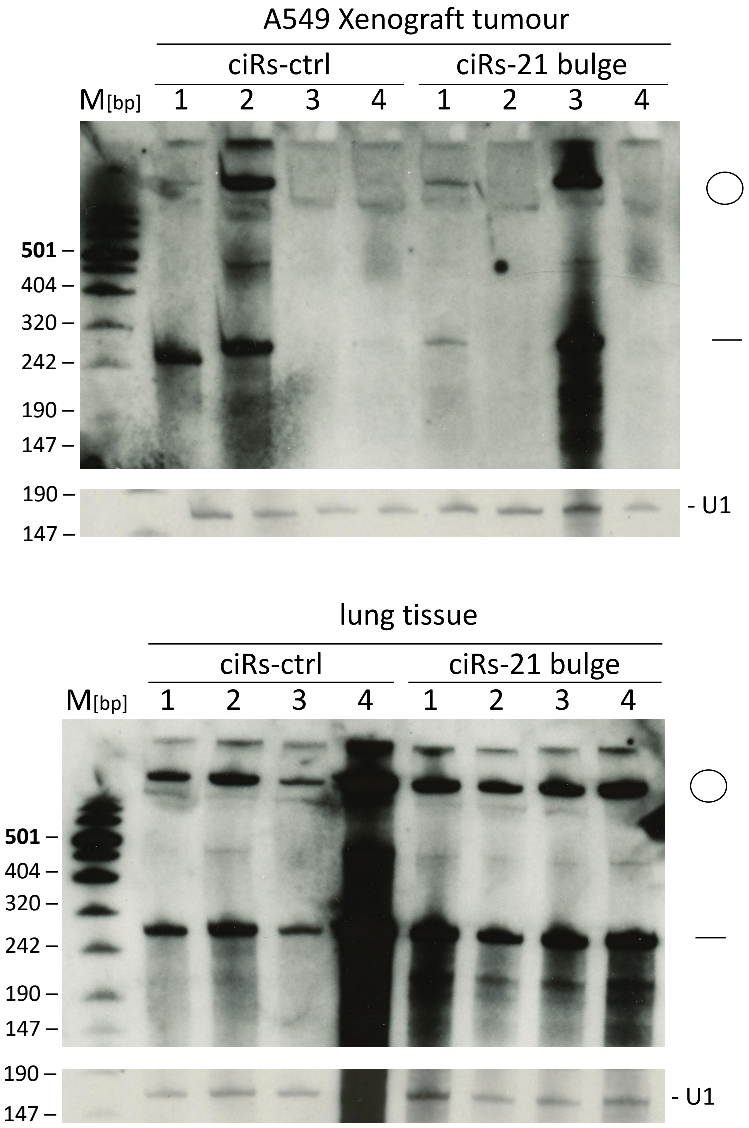

Supplement: zcaa014_Supplemental_Files [file zcaa014_supplemental_files.zip › NARC_Supplementary Figure Legends_R1.pdf]
